# Supplementary material for: Emplacement of the Argyle diamond deposit into an ancient rift zone triggered by supercontinent breakup
Source: Nat Commun. 2023 Sep 19;14:5274. doi: 10.1038/s41467-023-40904-8 (PMC10509175; doi:10.1038/s41467-023-40904-8)
Supplement: Supplementary file 1 — Supplementary Information [file 41467_2023_40904_MOESM1_ESM.pdf]

Supplementary Information to:

Emplacement of the Argyle diamond deposit into an ancient rift zone triggered by supercontinent breakup

Hugo K. H. Olierook, Denis Fougerouse, Luc Doucet, Yebo Liu, Murray J. Rayner, Martin Danišík, Daniel J. Condon, Brent I. A. McInnes, A. Lynton Jaques, Noreen J. Evans, Bradley J. McDonald, Zheng-Xiang Li, Christopher L. Kirkland, Celia Mayers, Michael T. D. Wingate

Compilation of previous emplacement ages of the Argyle AK1 lamproite

Three studies in the 1980s attempted to date emplacement of the Argyle lamproite via K-Ar (phlogopite), Rb-Sr (whole-rock and phlogopite) and Pb-Pb (sulfide, unpublished, Supplemental Table S2). With no data available for the latter, its reliability cannot be assessed. Three Rb-Sr studies[1, 2, 3] yielded either two-point isochrons of 1108 ± 9 Ma and 1181 ± 12 Ma or a 15-point isochron of 1159 ± 47 Ma with significant excess scatter MSWD = 7.3, p = 0; all ages recalculated using updated decay constants of [4]. A slightly older weighted mean K-Ar age of 1237 ± 26 Ma from three samples was also obtained[1], recalculated using the updated decay constants of [5]. The variation in dates can readily be explained by variable degrees of alteration. Pidgeon, Smith[1] recognized that the analyzed phlogopites had anomalously low K contents (~4 wt.%), half of what is expected of normal phlogopite, and clearly indicative of chloritization. This process induces mobility (both loss and gain) of Rb and K, and its radiogenic Ar and Sr products. At best, these dates can be considered minimum ages for the emplacement of the Argyle lamproite, but the K-Ar ages may also be affected by excess radiogenic Ar that could make the ages older.

Supplementary Table 1: Compilation of previously published geochronological data for the Argyle deposit.

Mineral/rock	Technique	Age ± 2σ (Ma)	MSWD	p	n	Reference	Comments/Interpretation
Partially chloritized phlogopite, whole-rock	Rb-Sr isochron	1126 ± 9	-	-	2 analyses	Skinner et al., 1985	Two-point isochron
Partially chloritized phlogopite, heavy mineral fraction (mostly apatite) from lamproite	Rb-Sr isochron	1201 ± 12	-	-	2 analyses	Sun et al., 1986	Two-point isochron, uncertainty assuming 1% error on $^{87}Rb/^{86}Sr$
Partially chloritized phlogopite	K-Ar weighted mean	1238 ± 26	2.6	0.07	3 of 3 analyses	Pidgeon et al., 1989	Anomalously low K content related to partial chloritization; potential excess Ar
Partially chloritized phlogopite, tuff, lamproite	Rb-Sr isochron	1178 ± 47	7.3	0	15 analyses	Pidgeon et al., 1989	Excess scatter; anomalously low K content related to partial chloritization

MODAL MINERALOGY & DIAMOND DISCOVERY

Supplementary Table 2: Modal mineralogy (by vol. %) determined via automated mineral analysis of two representative thin sections (a & b) of sample AK01-Lh01 from Argyle.

Mineral	Volume % of mineral	
**Primary phases / Dataset**	**AK1Lh01a**	**AK1Lh01b**
Chlorite	40.8	43.3
Quartz	40.6	37.1
Calcite	10.5	9.3
Titanite	3.7	3.6
Orthopyroxene-orthoamphibole	3.1	3.9
Ankerite	1.9	1.9
Plagioclase	0.40	0.25
Wollastonite	0.31	0.32
Augite	0.18	0.17
Olivine	0.13	0.10
Apatite	0.05	0.06
Chromite	0.04	0.01
K-feldspar/Sericite	0.02	0.02
Rutile	0.02	0.03
Dolomite	0.02	0.02
Kaolinite	0.017	0.005
Glue-resin	0.013	0.007
Zircon	0.007	0.006
Corundum/Gibbsite/Diaspore	0.009	0.002
Staurolite	0.007	0.003
Monazite	0.004	0.005
Biotite	0.002	0.003
Hornblende-Actinolite	0.001	0.002
Pyrite	0.002	0.001
Kyanite-Andalusite-Sillimanite	0.002	0.000
Allanite	0.001	0.001
Muscovite/Illite	0.000	0.000
Chalcopyrite	0.000	0.000
Ilmenite	0.000	0.000
Pyrrhotite	0.000	0.000
**The rest**	**0.001**	**0.001**
**Total**	**100**	**100**

Supplementary Fig. 1: Additional petrographic maps and observations from the Argyle deposit follows on the next two pages.

AK01-Lh01a, Full thin section petrographicand automated mineral analysis images

Transmitted, plane-polarized light

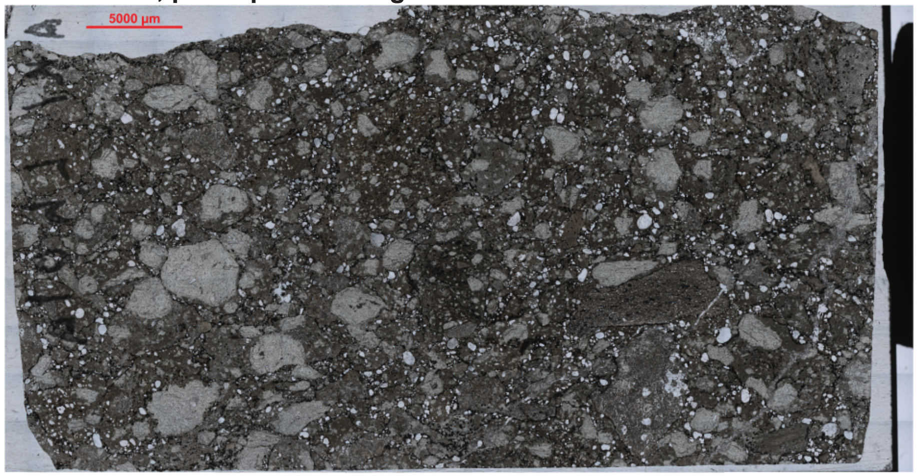

Backscattered electron image

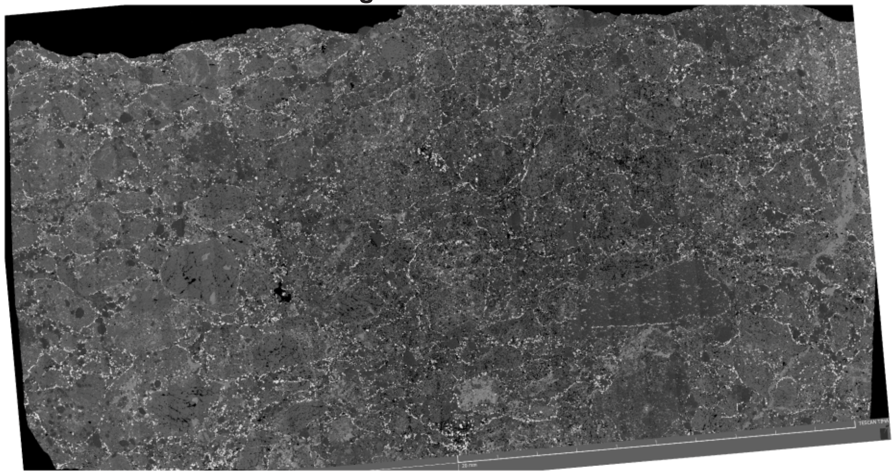

Transmitted, cross-polarized light

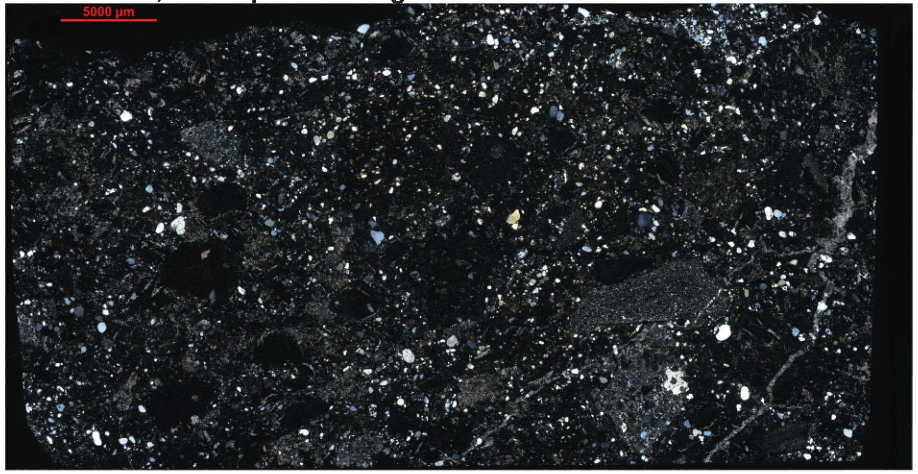

Automated mineral analysis map

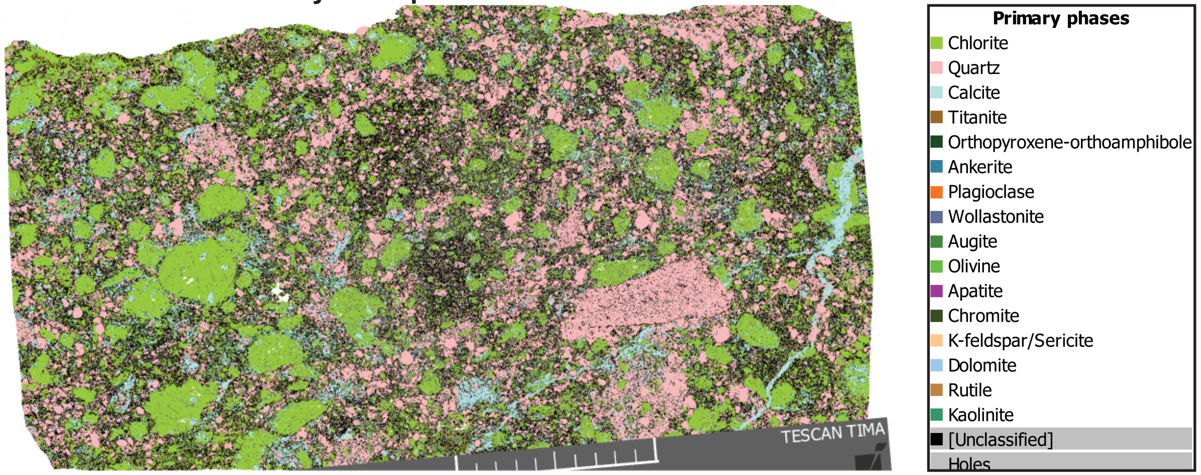

Reflected, plane-polarized light

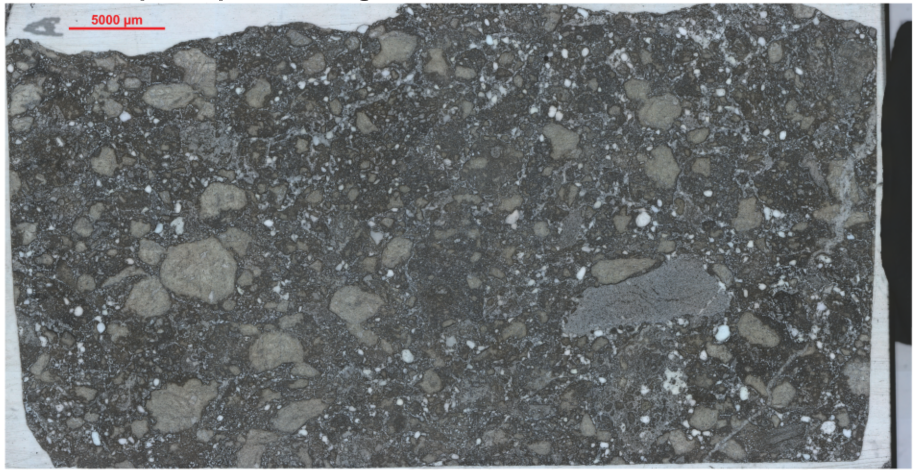

Ti Kα peak (predominantly highlighting titanite)

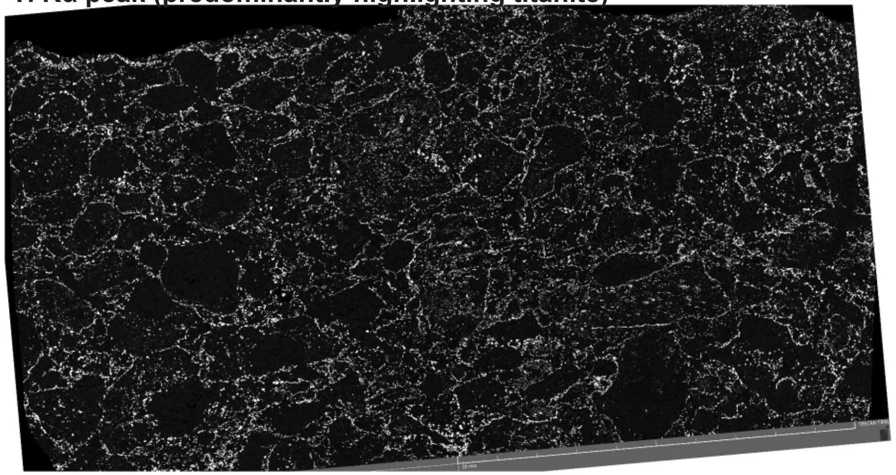

AK01-Lh01b, Full thin section petrographicand automated mineral analysis images

Transmitted, plane-polarized light

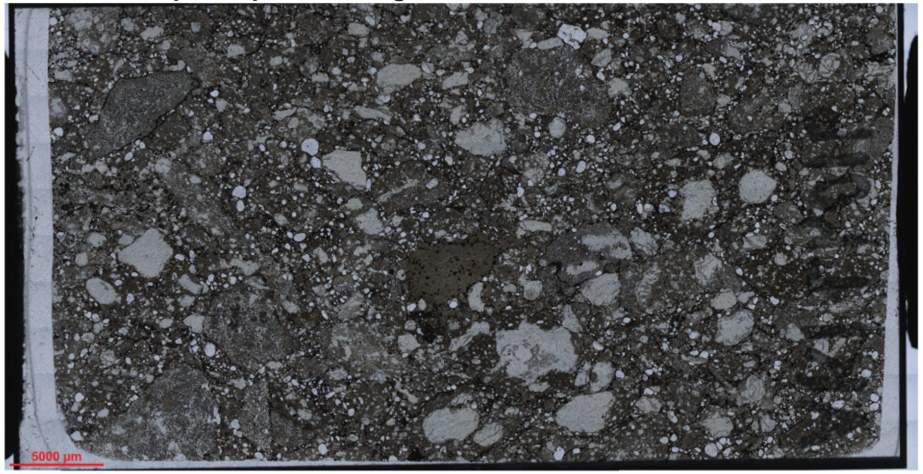

Backscattered electron image

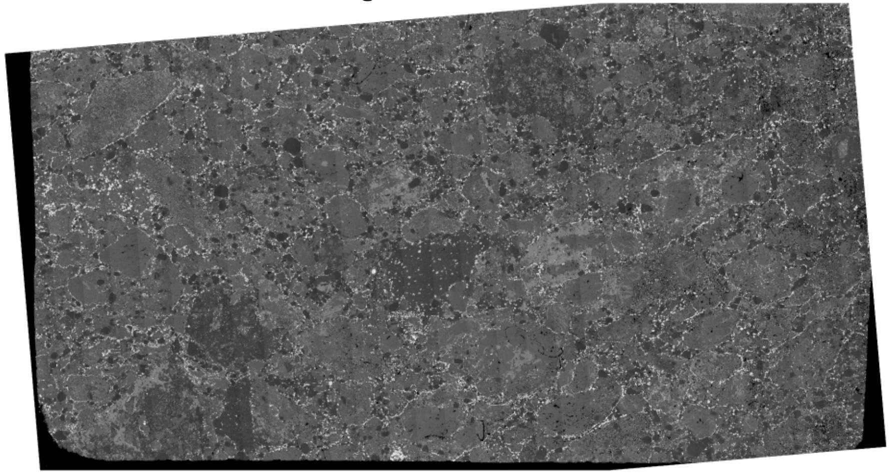

Transmitted, cross-polarized light

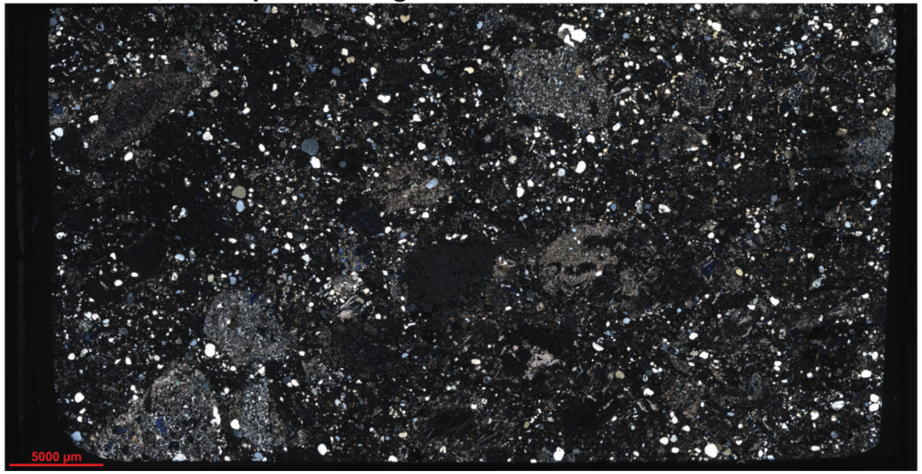

Automated mineral analysis map

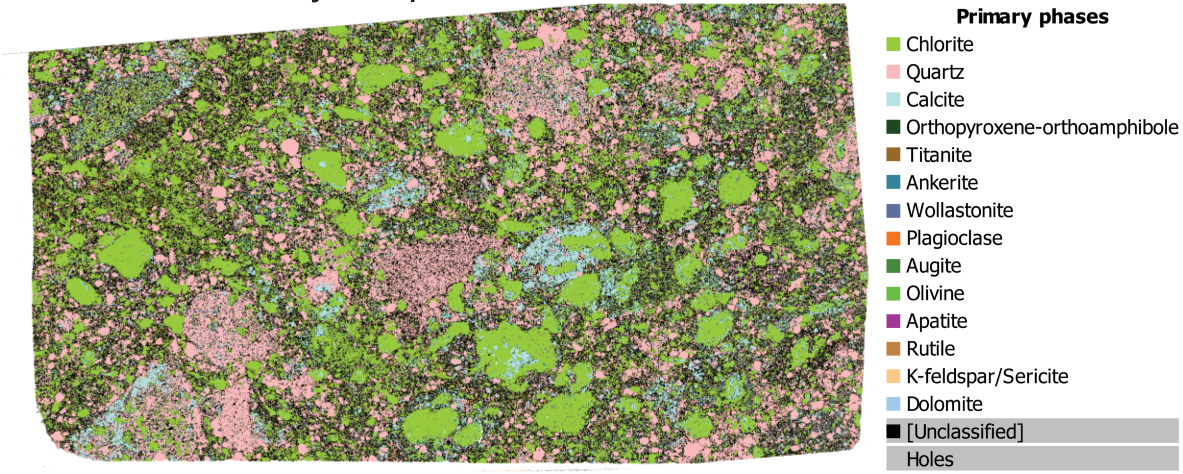

Reflected, plane-polarized light

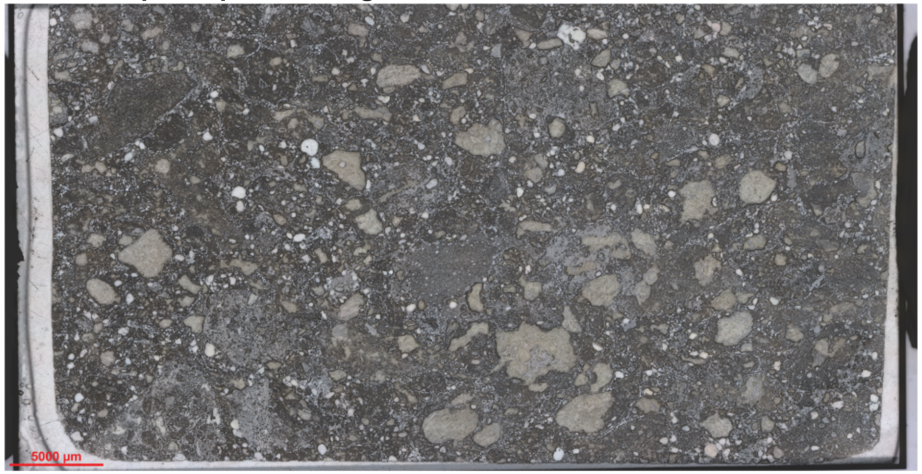

Ti Kα peak (predominantly highlighting titanite)

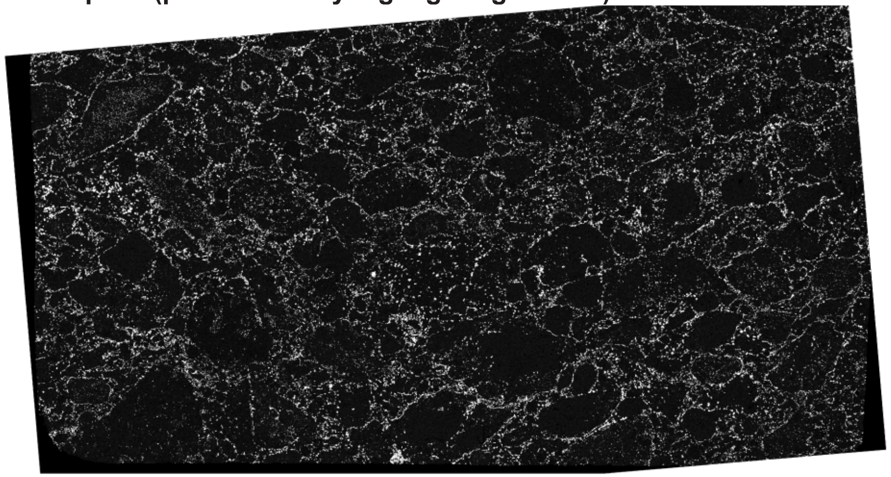

DISCOVERED DIAMOND WITHIN AK1-LH01

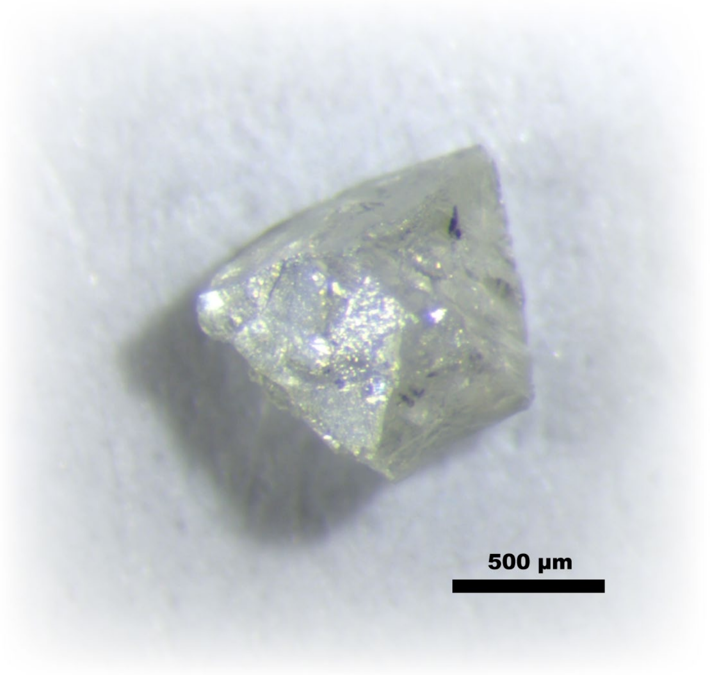

Supplementary Fig. 2: Dodecahedral diamond fragment discovered in AK1-Lh01.

Supplementary Fig. 3: Compendium of CL and BSE images for newly analyzed apatite (page 7), zircon (page 8 and 9) and titanite grains (page 10) follows on the next four pages.

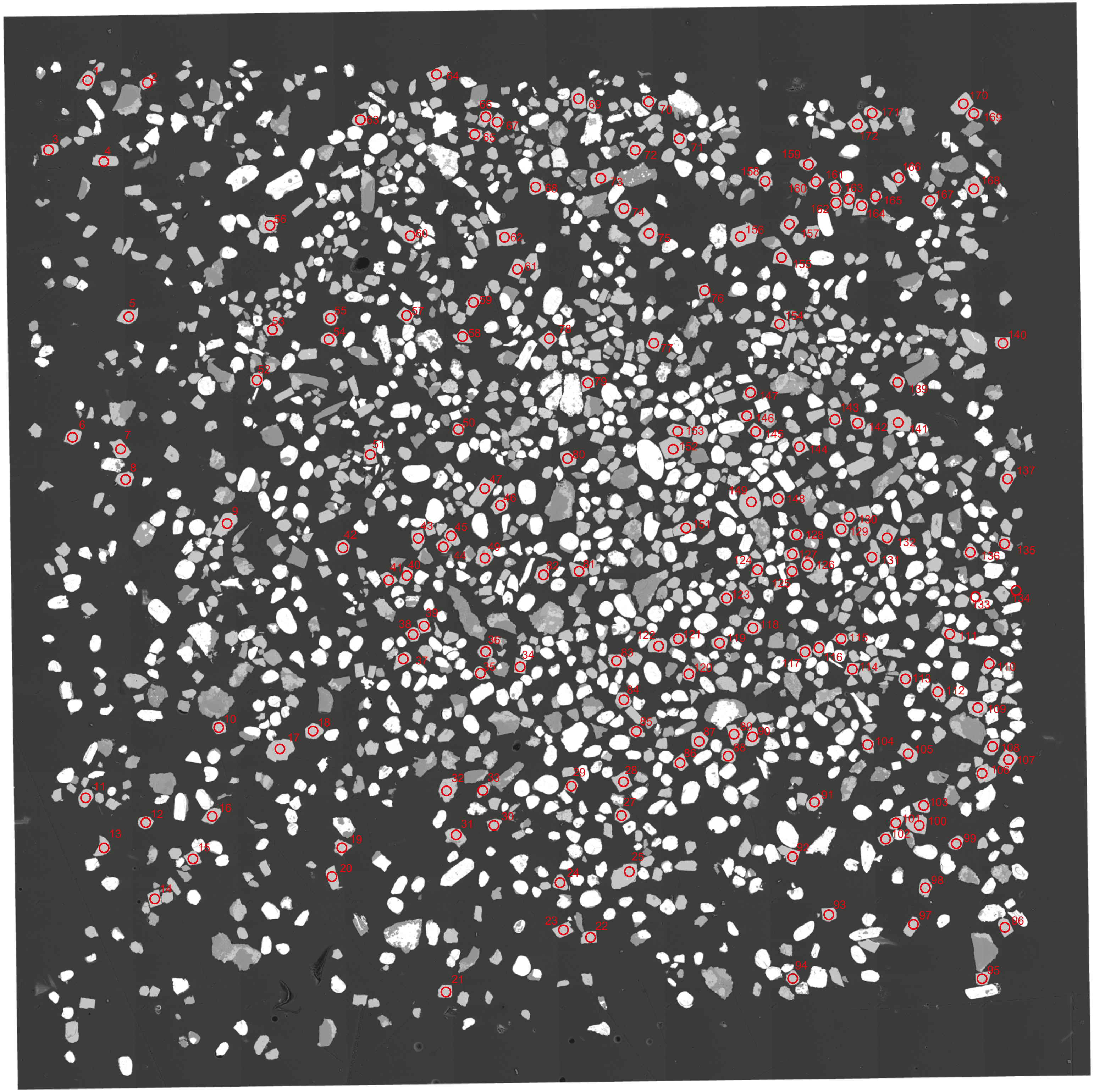

AK1-Lh01, Argyle apatite on Mount AK1-Lh01-zr+ap

BSE

22/06/2022, U-Pb LA-ICP-MS:
173 apatite analyses on
173 grains

apatite o1 50 um

Hugo Olierook

200 um

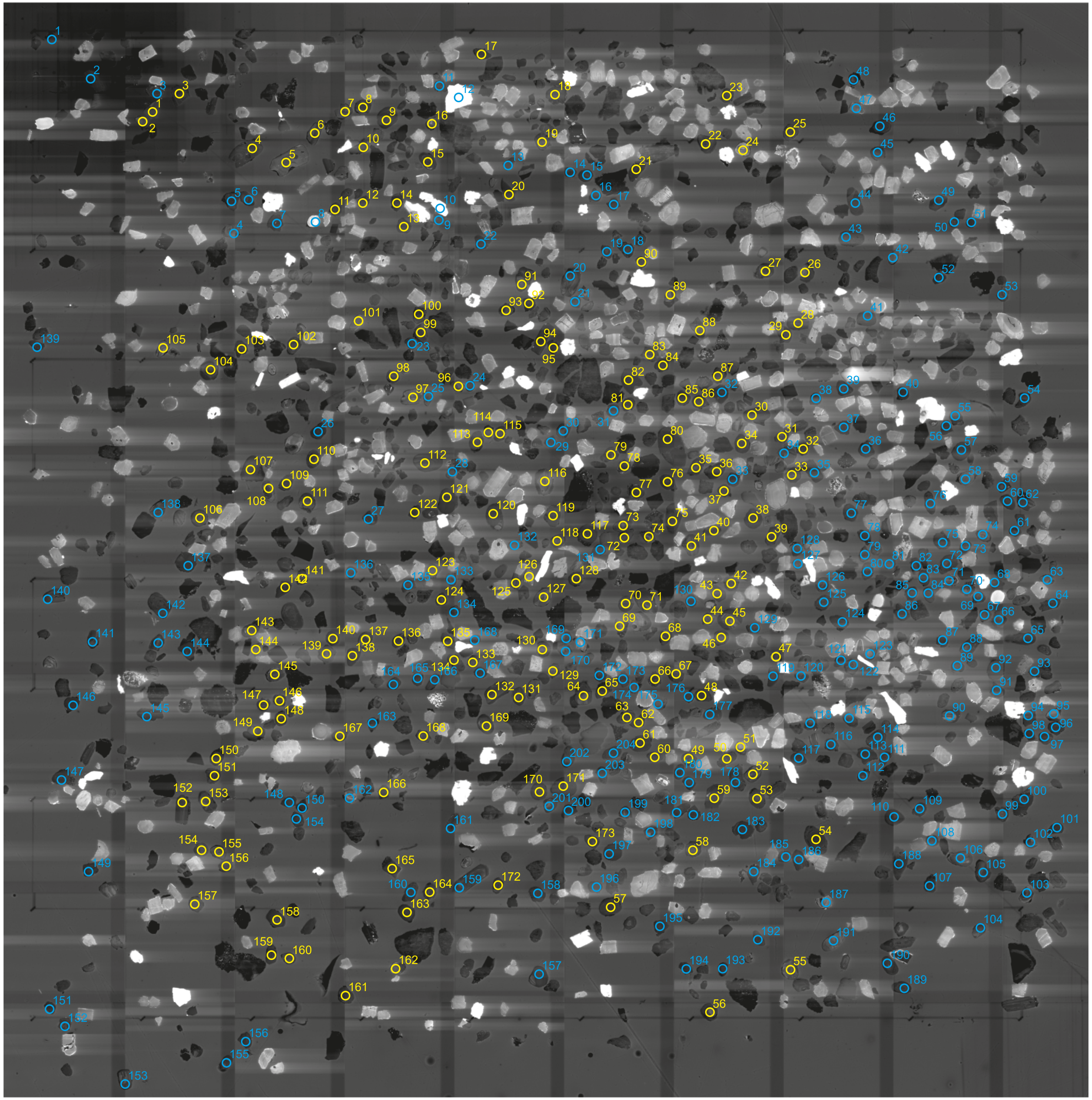

AK1-Lh01, Argyle
zircon on Mount
AK1-Lh01-zr+ap

CL

25/08/2022, U-Pb LA-ICP-MS:
173 zircon analyses on
173 grains

zircon, part 1 o1 30 um

1/09/2022, U-Pb LA-ICP-MS:
204 zircon analyses on
204 grains

zircon, part 2 o1 30 um

Hugo Olierook

200 um

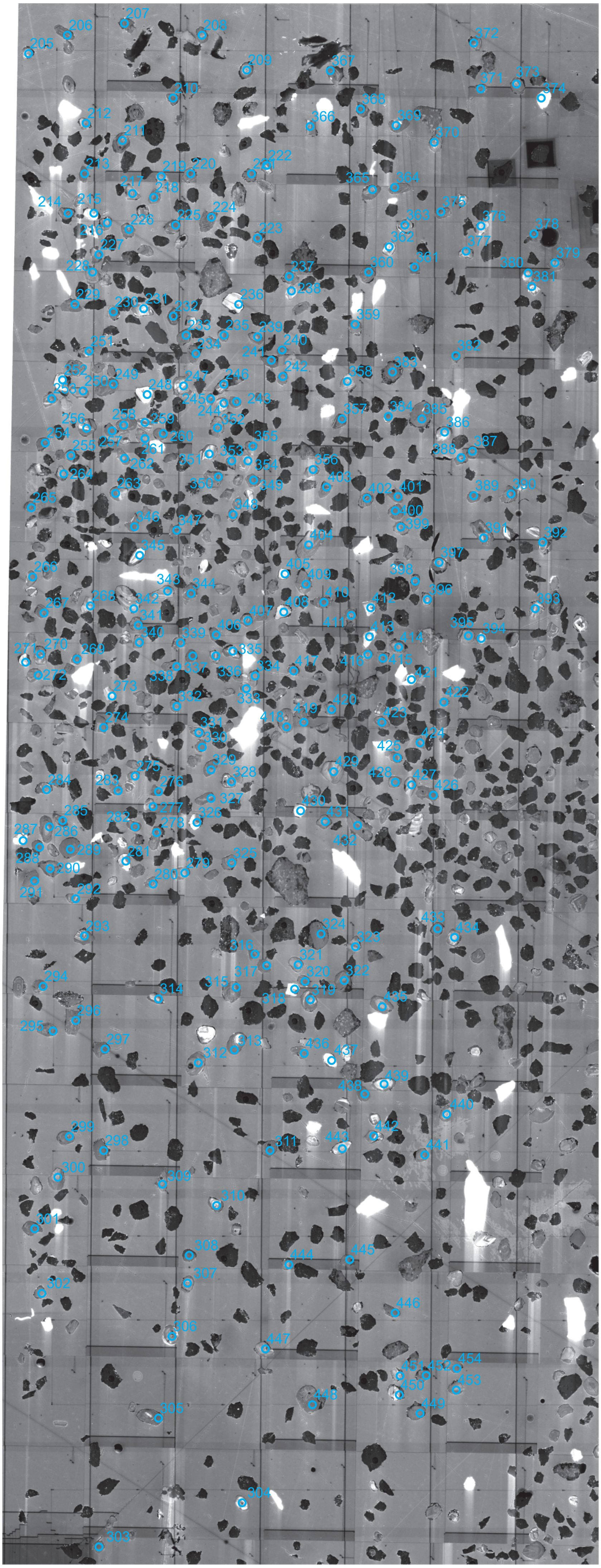

AK1-Lh01, Argyle , zircon on Mount AK1-Lh01-H3.3NM1.7

CL

01/09/2022, U-Pb LA-ICP-MS:
250 zircon analyses on
250 grains

zircon, part 2 ₒ1 30 um

Note: 454 analyses total
on two mounts for day 2

200 um

Hugo Olierook

BSE

AK1-Lh01, Argyle , titanite
on Mount AK1-Lh01-H3.3NM1.7

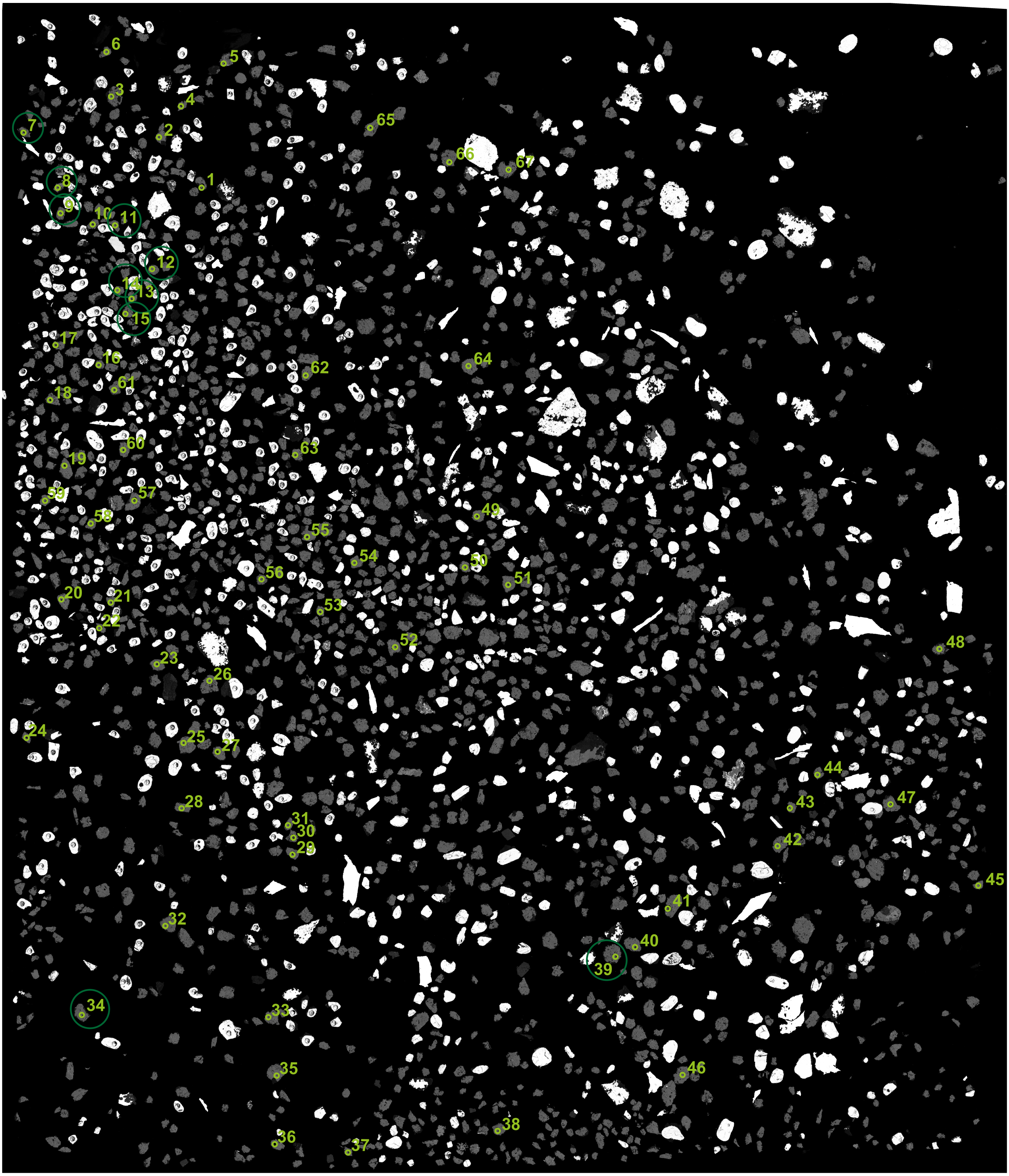

May 2023, U-Pb ID-TIMS
12 entire titanite grains

Hugo Olierook

12/09/2022, U-Pb LA-ICP-MS:
67 titanite analyses on
67 grains

titanite 1 30 um

200 um

ADDITIONAL RESULTS

Reliability of youngest zircon analyses

Given the paucity of young dates, and the importance of establishing a maximum depositional age, the reliability of the three c. 1360–1300 Ma zircon dates should be closely examined. First, the laser ablation spectra of the two c. 1310 Ma grains show normal profiles for Pb and U isotopes with no inclusions, no core–rim relationships and no ablation through parts of the surrounding resin (Supplementary Fig. S2). The slightly older grain shows a steep gradient indicative of minor Pb loss, and a Th-rich inclusion. Second, both younger grains show that oscillatory-zoning is truncated by the crystal edges, indicating sedimentary transport of originally magmatic grains, consistent with a detrital origin for these zircon grains (Fig. 3b). Third, contamination, although always a possibility, is extremely unlikely as it was several weeks prior the processing of our Argyle sample that any samples with ages of ca. 1310 Ma were processed in the mineral separation facility in the JdLC. Thus, we consider the age of 1311 ± 9 Ma to be a robust maximum emplacement age for the Argyle lamproite.

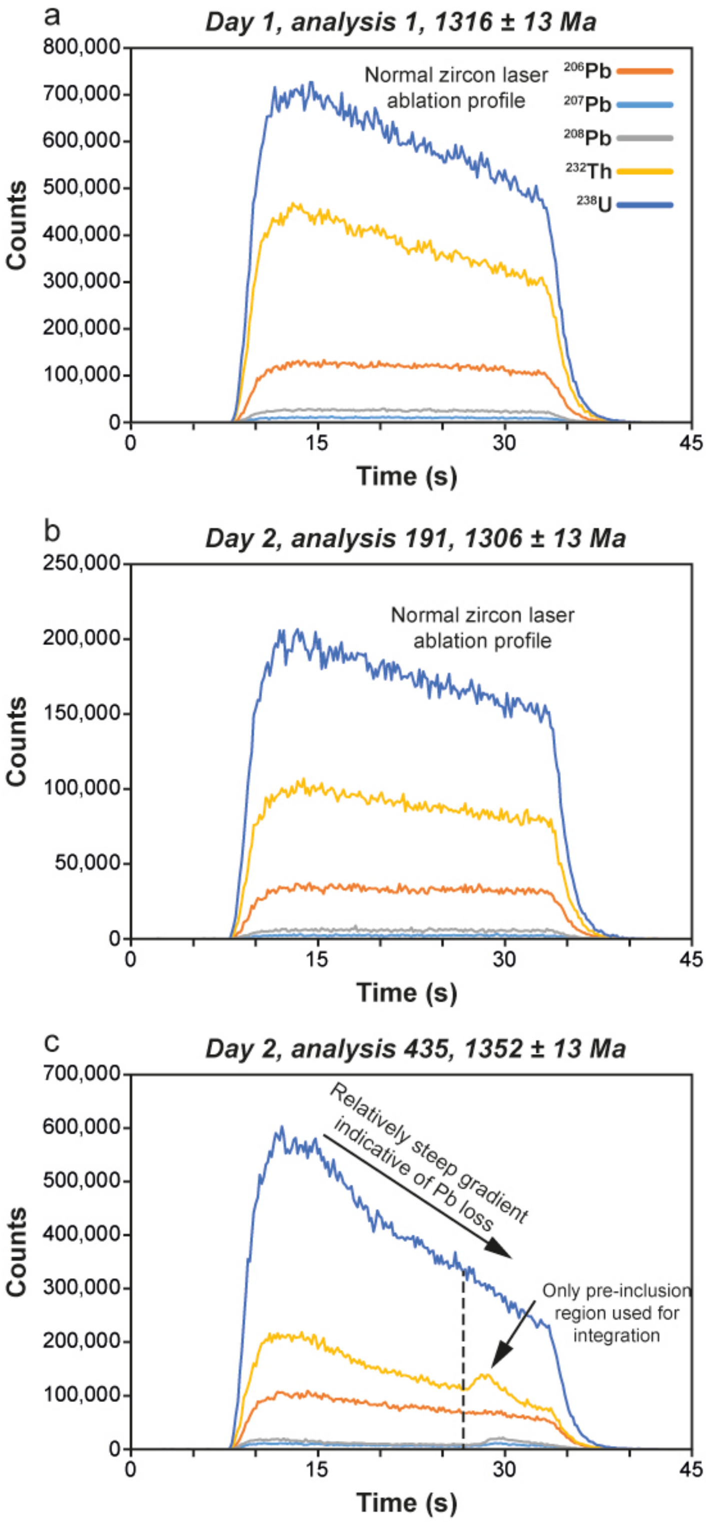

Supplementary Fig. 4: Laser ablation profiles for the three youngest detrital zircon grains. (a & b) The two youngest analyses show robust profiles. (c) The older analysis shows a stronger decline, consistent with minor Pb loss, and an inclusion towards the end of the ablation. Note that ^{202}Hg, ^{204}Pb and ^{235}U, which were also collected, are not shown for clarity due to their low abundances (<2000 counts per time slice).

Apatite and zircon (U-Th)/He geochronology

See main text for interpretation.

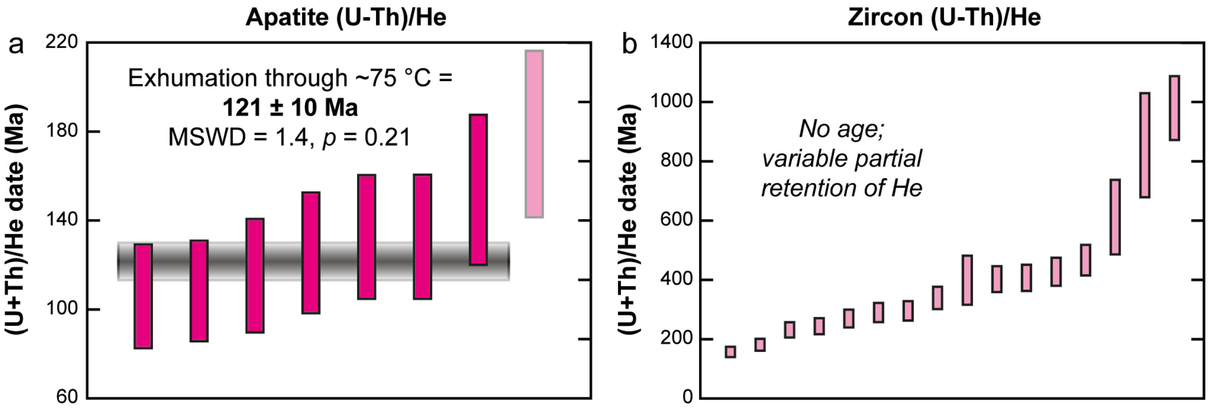

Supplementary Fig. 5: (U-Th)/He data of the Argyle lamproite. (a) Weighted mean plot of detrital apatite. (b) Weighted mean plot of detrital zircon.

SUPPLEMENTARY REFERENCES

1. Pidgeon RT, Smith CB, Fanning CM. Kimberlite and lamproite emplacement ages in Western Australia. *Kimberlites and related rocks* **1**, 382-391 (1989).
2. Sun S-S, Jaques AL, McCulloch MT. Isotopic evolution of the Kimberley block, Western Australia. *International Kimberlite Conference* **4**, 346-348 (1986).
3. Skinner EMW, Bristow JW, Smith CB, Scott Smith BH, Dawson JB. Proterozoic kimberlites and lamproites and a preliminary age for the Argyle lamproite pipe, Western Australia. *Trans Geol Soc S Afr* **88**, 335-340 (1985).
4. Villa IM, De Bièvre P, Holden NE, Renne PR. IUPAC-IUGS recommendation on the half life of ^{87}Rb. *Geochim Cosmochim Acta* **164**, 382-385 (2015).
5. Renne PR, Balco G, Ludwig KR, Mundil R, Min K. Response to the comment by W.H. Schwarz et al. on "Joint determination of ^{40}K decay constants and ^{40}Ar*/^{40}K for the Fish Canyon sanidine standard, and improved accuracy for ^{40}Ar/^{39}Ar geochronology" by PR Renne et al. (2010). *Geochim Cosmochim Acta* **75**, 5097-5100 (2011).
