# Supplementary material for: Emplacement of the Argyle diamond deposit into an ancient rift zone triggered by supercontinent breakup
Source: Nat Commun. 2023 Sep 19;14:5274. doi: 10.1038/s41467-023-40904-8 (PMC10509175; doi:10.1038/s41467-023-40904-8)
Supplement: Supplementary file 3 — Description of Additional Supplementary Files [file 41467_2023_40904_MOESM3_ESM.pdf]

Description of Additional Supplementary Files

Supplementary Data 1: Reference materials for geochronological data, including U-Pb apatite, U-Pb zircon and U-Pb titanite.

Supplementary Data 2: Full geochronological data for AK01- Lh01, including U-Pb apatite, U-Pb zircon, U-Pb titanite, (U+Th)/He apatite and (U+Th)/He zircon.
